# Resistance to the Plant Defensin NaD1 Features Modifications to the Cell Wall and Osmo-Regulation Pathways of Yeast

**DOI:** 10.3389/fmicb.2018.01648

**Published:** 2018-07-24

**Authors:** Amanda I. McColl, Mark R. Bleackley, Marilyn A. Anderson, Rohan G. T. Lowe

**Affiliations:** Department of Biochemistry and Genetics, La Trobe Institute for Molecular Science, La Trobe University, Melbourne, VIC, Australia

**Keywords:** antifungal, defensin, genome, yeast, resistance, NaD1, cell wall, stress

## Abstract

Over the last few decades, the emergence of resistance to commonly used antifungal molecules has become a major barrier to effective treatment of recurrent life-threatening fungal diseases. Resistance combined with the increased incidence of fungal diseases has created the need for new antifungals, such as the plant defensin NaD1, with different mechanisms of action to broaden treatment options. Antimicrobial peptides produced in plants and animals are promising new molecules in the arsenal of antifungal agents because they have different mechanisms of action to current antifungals and are often targeted specifically to fungal pathogens (van der Weerden et al., 2013). A key step in the development of novel antifungals is an understanding of the potential for the fungus to develop resistance. Here, we have used the prototypic plant defensin NaD1 in serial passages with the model fungus *Saccharomyces cerevisiae* to examine the evolution of resistance to plant antifungal peptides. The yeast strains did develop tolerance to NaD1, but it occurred more slowly than to the clinically used antifungal caspofungin. Sequencing the genomes of the strains with increased tolerance failed to identify any ‘hotspot’ mutations associated with increased tolerance to NaD1 and led to the identification of 12 genes that are involved in resistance. Characterization of the strains with increased tolerance to NaD1 also revealed changes in tolerance to abiotic stressors. Resistance developed slowly via an accumulation of single nucleotide mutations and had a fitness penalty associated with it. One of the genes identified *FPS1*, revealed that there is a common mechanism of resistance to NaD1 that involves the osmotic stress response pathway. These data indicate that it is more difficult to generate resistance to antimicrobial peptides such as NaD1 compared to small molecule antifungals.

## Introduction

Pathogenic fungi have become a serious threat to both agriculture and human health (Murray and Brennan, 2009). In human health, fungal pathogens are detrimental to immunocompromised individuals, such as individuals with HIV, transplant recipients and cancer patients receiving chemotherapy (Ortega et al., 2010). Indeed, some invasive fungal diseases can become life-threatening in the immunocompromised and mortality can reach up to 80% (Lass-Flörl, 2009). There are very few therapeutic options for systemic fungal infections, and some fungicides are known to be dangerous to human health due to severe side effects such as toxicity (Ortega et al., 2010). Fungicide resistance occurs when a fungal pathogen becomes less susceptible to an antifungal agent. Resistance is broadly characterized by the mechanism by which it occurs. These mechanisms include; alteration of the target site in a protein, detoxification of the fungicide, overexpression of the target site, and the use of efflux pumps to expel the fungicide (Leroux et al., 2002). The increased use of the small molecule antifungal drugs that are currently in the clinic as well as related molecules used in agriculture has led to reports of fungal pathogens resistant to almost all common antifungals (Verweij et al., 2009). There is a need for new antifungal agents to battle the phenomenon of fungal resistance; antifungal proteins are one attractive option for development (Sanglard et al., 1996; van der Weerden et al., 2013).

A wide variety of organisms produce antifungal peptides as part of their innate immunity arsenal (van der Weerden et al., 2013). They are highly represented in plants where defensins are the largest family. Plant defensins are small proteins of 45 to 54 amino acids that are ubiquitous in the seeds, leaves and flowers of all plants examined (Berkut et al., 2014). They are usually produced constitutively as a defense against pathogens, particularly in reproductive tissues and seeds (Lay et al., 2003). They are also expressed in response to infections and environmental stress (Lay et al., 2003; Sagaram et al., 2011). There are thousands of plant defensins in public sequence databases. They share a common structure, but are highly variable in sequence and, not surprisingly, they often have different mechanisms of action (Parisi et al., 2018). The mechanism of action of only a handful of defensins has been elucidated. They often have multistep mechanisms that affect more than one target in the fungus (Parisi et al., 2018). Hence, it is expected that resistance to defensins is likely to develop more slowly than resistance to smaller antifungal molecules that interact with a single site, composed of a few amino acids, on a single protein target. NaD1 is a potent antifungal defensin that accumulates in the flowers of the ornamental tobacco plant *Nicotiana alata*, where it functions to protect the reproductive organs from damage by fungal pathogens (Lay et al., 2003). NaD1 has a well-characterized structure, and several features of its mechanism of action have been well described but not completely elucidated (Lay et al., 2012). NaD1 has at least a three-step mechanism of action that involves: interaction with the fungal cell wall (van der Weerden et al., 2008), movement across the plasma membrane, induction of oxidative stress, and interaction with phosphatidylinositol 4,5 bisphosphate. These processes lead to damage of the inner leaflet of the cell membrane and cell death within 10 min of exposure to NaD1 (van der Weerden et al., 2010; Hayes et al., 2014; Poon et al., 2014; Payne et al., 2016).

In this study, yeast strains were generated that have increased tolerance to NaD1, and genetic mutations linked to the decreased response to NaD1 were identified. Phenotypic characterization of resistant lines revealed slower growth rates, as well as cell wall changes reflected as sensitivity to the anionic detergent SDS and the chitin binding molecule calcofluor white (CFW). That is, there was a fitness trade-off associated with NaD1-resistance. Mutations across twelve genes correlated with NaD1 resistance. These genes were associated with diverse aspects of cellular processes suggesting that NaD1 acts upon multiple cellular targets. Affected locations or processes included the cell wall, transporters and signaling pathways. Mutations in the gene *FPS1* indicate glycerol accumulation may modulate NaD1 antifungal activity. Resistance to NaD1 occurred more slowly than resistance to caspofungin in similar experiments.

## Materials and Methods

### Fungal Strains

The *S. cerevisiae* strain BY4741 (*MAT*α*his3*Δ*0 leu2*Δ*0 met15*Δ*0 ura3*Δ*0*) was purchased from Thermo Scientific. Single deletion strains were retrieved from the haploid non-essential deletion collection (Thermo Scientific) (Winzeler et al., 1999). *S. cerevisiae* was routinely cultured on YPD-Agar (1% yeast extract, 2% peptone, 2% dextrose, 2% agar) medium at 30°C.

### Antifungal Molecules

NaD1 and NaD2 were purified from *Nicotiana alata* flowers as described in Lay et al. (2003) and Dracatos et al. (2014). HXP4 and DmAMP1 were expressed in *Pichia pastoris* and purified as described previously (Hayes et al., 2013; Bleackley et al., 2016). CP29 was purchased from GL Biochem (China), BPTI (synonym Aprotinin) was purchased from Astral Scientific (Australia), caspofungin was purchased from Sigma (Australia).

### Culturing in the Presence of Antifungal Molecules to Develop Resistance

*S. cerevisiae* BY4741 was grown overnight at 30°C with agitation in 5 mL of YPD. The overnight culture was then diluted to an OD 600 nm of 0.01 in 50% strength PDB medium (½ PDB) before addition of antifungal molecules. Cultures were initially grown with the antifungal molecules at 0.5x the minimum inhibitory concentration (MIC) or 1x MIC alongside a negative control lacking antifungals. Three independent lines for the test and controls were grown at the same time. The cultures were incubated overnight at 30°C with agitation. The cultures that exhibited growth at the highest concentration of the antifungal molecules were sub-cultured with medium containing a higher concentration of the antifungal molecule. Sub-culturing was stopped once growth occurred at 32 times the original MIC.

### Single-Colony Isolation of Resistant Strains

Cultures that were more tolerant to the antifungal molecule were streaked out for single colonies on non-selective YPD agar. Three colonies were picked from each line, and their resistance was re-tested. The colony with the highest resistance to the antifungal was retained for further experimentation. The MIC of pure strains isolated from each culture was broadly equivalent (Supplementary Figure 1).

### Antifungal Assay

Antifungal assays were performed as described in Hayes et al. (2013). Briefly, cultures were grown overnight (30°C, 250 rpm) in 5 mL YPD and diluted to an OD600 of 0.01 in ½ PDB. Antifungal molecules were prepared at 10x the assay concentration, and 10 μL was mixed with 90 μL of diluted yeast culture before incubation for 24 h at 30°C. The final OD600 was measured using a SpectraMAX M5e plate reader (Molecular Devices).

### Cell Growth Assay

*S. cerevisiae* BY4741 cultures were grown overnight (30°C, 250 rpm) in 5 mL of YPD and diluted to an OD_600_ of 0.5 in 1 mL YPD and ½ PDB. Each culture (100 μL) was incubated in a SpectraMAX M5e plate reader (Molecular Devices) at 30°C in a 96-well microtiter plate format. Optical density at 600 nm was recorded every 30 min over the 48 h culture period.

### Cell Size and Area Measurement

*S. cerevisiae* BY4741 cultures were grown overnight (30°C, 250 rpm) in 5 mL of YPD and were imaged using an Olympus IX81 brightfield microscope (LIMS Bioimaging Facility). Cell dimensions were measured from images using FIJI software (Schindelin et al., 2012). A minimum of 30 cells was measured for each sample.

### Stress Assay With Hydrogen Peroxide, Calcofluor White, NaCl, and SDS

YPD agar medium (25 mL) was amended to a final concentration of hydrogen peroxide (0.625 mM, 1.25 mM, 2.5 mM, 5 mM), CFW (1 μg/mL, 2.5 μg/mL, 5 μg/mL, 10 μg/mL), NaCl (100 mM, 200 mM, 300 mM), or SDS (12.5 μg/mL, 25 μg/mL, 50 μg/mL, 100 μg/mL) just before each plate was poured. Yeast cultures were grown overnight in 5 mL of YPD before dilution to an OD 600 nm of 0.1. A fivefold dilution series of each culture was spotted onto the plate (4 μL per spot) and incubated overnight at 30°C before being photographed.

### Stress Assay With Ultraviolet Light

*S. cerevisiae* cultures were grown overnight in 5 mL YPD and diluted to an OD 600 nm of 0.1 in 1 mL MilliQ-purified water. A fivefold dilution series of each strain (4 μL per spot) was added to the YPD agar plate and allowed to dry, before exposure to UV light (Phillips, 30 W bulb at 50 cm) for 1.2, 2.4, 5.2, or 10.4 min.

### Stress Assay With Heat

*S. cerevisiae* cells were grown overnight in 5 mL YPD and diluted to an OD 600 nm of 0.1 in 1 mL MilliQ-purified water. Diluted cultures (100 μL) were heated (30°C, 37°C, 41°C, or 46°C) for 30 min. Survival was assessed after heat treatment using a spot assay on YPD agar.

### DNA Extraction From Wild-Type and Resistant Strains of *S. cerevisiae*

Genomic DNA was extracted using the Qiagen DNeasy^®^ plant miniprep kit. Three individual lines of NaD1-resistant strains and three lines of the no-treatment controls were sequenced. Sequencing was completed at the La Trobe Genomics Platform, using Illumina MiSeq V3 chemistry. One run was performed for all six genomes, generating 25 million 300 bp paired-end reads. The pre-processing and variant discovery steps were performed as described by the GATK best practices and are summarized in McKenna et al. (2010).

### Genomic Analysis of Resistant Strains of *S. cerevisiae* Sequence Pre-processing

Picard tools (v.2.4.1) fastqtosam was used to convert raw sequence files into Sam format and to add read group information. Any Illumina adapters were identified and marked using Picard (v.2.4.1) markilluminaadapters. BWA-mem (v.0.7.12) was used to align reads to the reference *S. cerevisiae* (R64-1-1.23) genome (Engel et al., 2014). Alignment files were merged, and duplicate reads were marked using Picard (v.2.4.1) mergebamalignment and markduplicates. Local alignments were optimized, and sequence quality scores were recalibrated using GATK (v.3.6) realignertargetcreater and baserecalibrator.

### Variant Discovery

GATK (v.3.6) Haplotypecaller was used to find genome variations that were either SNVs (single-nucleotide variants) or INDELs (insertion/deletion) simultaneously, also using known variants from dbSNP (Sherry et al., 2001). The samples were merged using GATK (v.3.6) combinegvcf, and then GenotypeGVCFs was used to rescore and genotype the combined gVCFs. GATK (v.3.6) VariantFiltration and VariantRecalibrator were used to extract SNVs and indels from the combined call set based on the default quality parameters, the SNVs and indels were then labeled as passed or filtered.

### Variant Refinement

The high-quality variants identified during the variant discovery process were annotated using SnpEff (v.2.4) (Cingolani et al., 2012). SnpEff was used to determine whether each mutation was predicted to alter an encoded protein sequence (**Table 3**). Variant effect predictor (VEP) marked any codon changes as either tolerant or deleterious (McLaren et al., 2016). SnpSift (v.2.4) was used to was used to identify SNVs or indels that were present in NaD1 resistant replicates and not in the Control strains (**Table 3**). The variants selected during refinement were inspected manually using IGV (v.2.3.77) to rule out unexpected processing artifacts (Robinson et al., 2011).

### Sanger Sequencing of the FKS1 Gene of Caspofungin-Resistant Mutants

The *FKS1* gene from three individual lines of caspofungin-resistant strains and a no treatment control was amplified by PCR using primers TCAAGGAAGGCAAGAAAAGCTA and GAGGCCGATACTGGTGAAAA and NEB Q5 proofreading polymerase according to the manufacturer’s directions. Initial denaturation was at 95°C for 2 min, followed by 30 cycles of: 95°C 30 s, 55°C 30 s, 72°C 2 min, and a final extension at 72°C 2 min. Sanger sequencing of the FKS1 amplicon using primers “TCAAGGAAGGCAAGAAAAGCTA” and “CTGCATTTGCCCCTCTACAT” was completed by the Australian Genome Research Facility (AGRF). Sequence data were analyzed using Geneious software.

## Results

### Evolution of Resistance to NaD1

**FIGURE 1:**
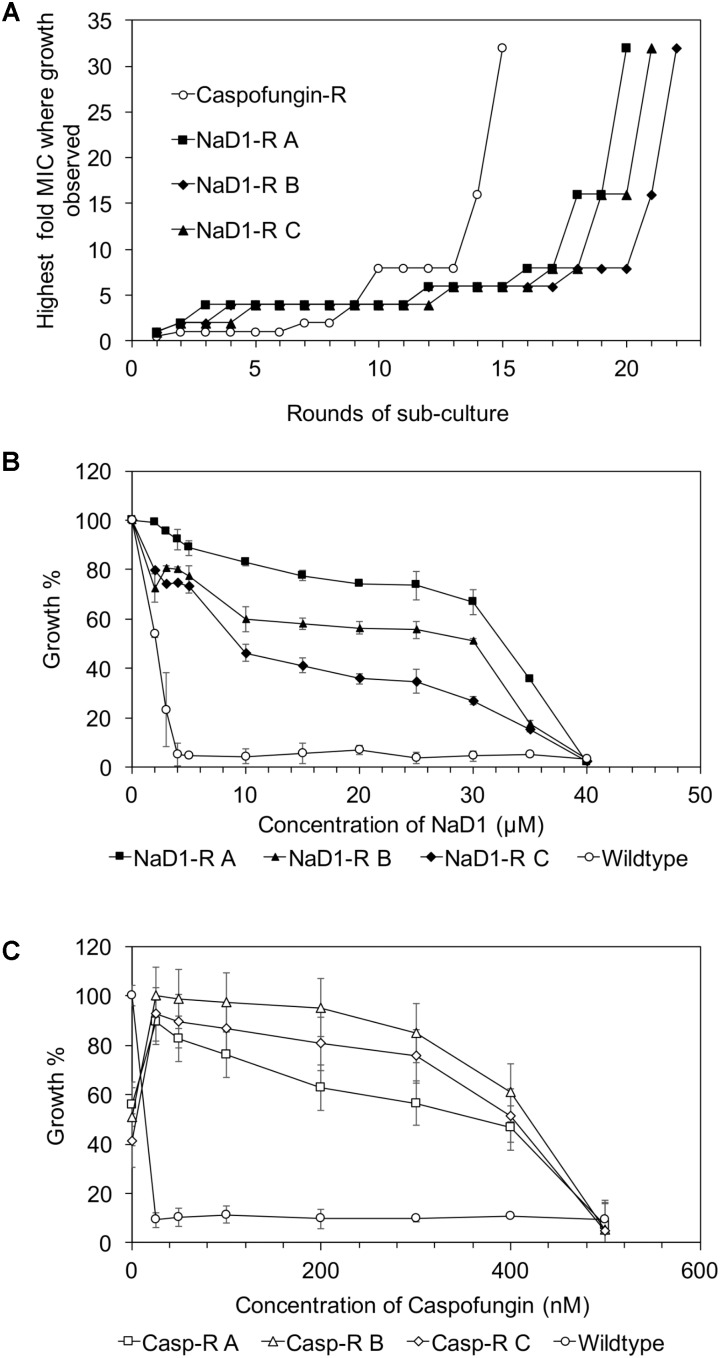
Mild resistance to NaD1 or caspofungin can be evolved *in vitro.* Summary of the development of resistance during sub-culturing in the presence of Caspofungin or NaD1 **(A)**. Three independent strains of NaD1-resistant yeast are shown, along with a representative example of the caspofungin-resistant lines. The antifungal activity of NaD1 **(B)** and caspofungin **(C)** against NaD1-resistant and caspofungin-resistant lines, respectively, is graphed relative to the highest measured OD for each strain. Error bars represent ±standard error of the mean (*n* = 3).

Yeast strains with increased tolerance to NaD1 or caspofungin were developed by continuous culture of *S. cerevisiae* in sub-lethal concentrations of each antifungal molecule. Each time the MIC increased, the dose of antifungal was doubled. The starting concentration of NaD1 was 1 μM; it took 20 rounds of sub-culturing for NaD1-R A, 21 rounds for NaD1-R C and 22 rounds for NaD1-R B to achieve growth in 32 μM NaD1 (**Figure 1A**). In contrast, it took only 15 rounds of sub-culture to achieve growth in caspofungin at concentrations 32-fold higher than the initial MIC 10 nM (**Figure 1A**).

Three genetically pure strains of each of the NaD1-resistant and caspofungin-resistant lines were isolated, and their resistance phenotype was confirmed using a standard antifungal growth assay. The colony with the most resistance for each line was used for all further experimentation. The NaD1-resistant isolates were 10-fold more resistant to NaD1 than the no-treatment control lines that had been passaged at the same time, with an MIC of 40 μM compared to an MIC of 4 μM (**Figure 1B** and **Table 1**). The caspofungin-resistant isolates were 25-fold more resistant to caspofungin with an MIC of 500 nM compared to the no treatment control which had an MIC of 20 nM (**Figure 1C** and **Table 1**). In most fungal species, resistance to caspofungin occurs via mutations to the FKS1 gene within a “hot spot” zone affecting residues Phe639 to Pro647 (Katiyar and Edlind, 2009). Sequencing of the entire FKS1 gene of our caspofungin-resistant strains revealed that all three strains contained a single point mutation (F639V) confirming resistance was derived by the most commonly observed mechanism (Supplementary Figure 2).

**Table 1:** The MIC of NaD1- and caspofungin-resistant lines of *S. cerevisiae*.

Strain	NaD1 MIC (μM)	Caspofungin MIC (nM)
Wild-type	4	20
NaD1-R A	40	25
NaD1-R B	40	25
NaD1-R C	40	25
Caspofungin-R A	4	500
Caspofungin-R B	4	500
Caspofungin-R C	4	500


*The minimum inhibitory concentration (MIC) of NaD1 and caspofungin are summarized for NaD1 resistant isolates, caspofungin resistant isolates and the parental wild type line of S. cerevisiae.*

### Resistance to NaD1 Confers Resistance to Some but Not All Antifungal Peptides

**FIGURE 2:**
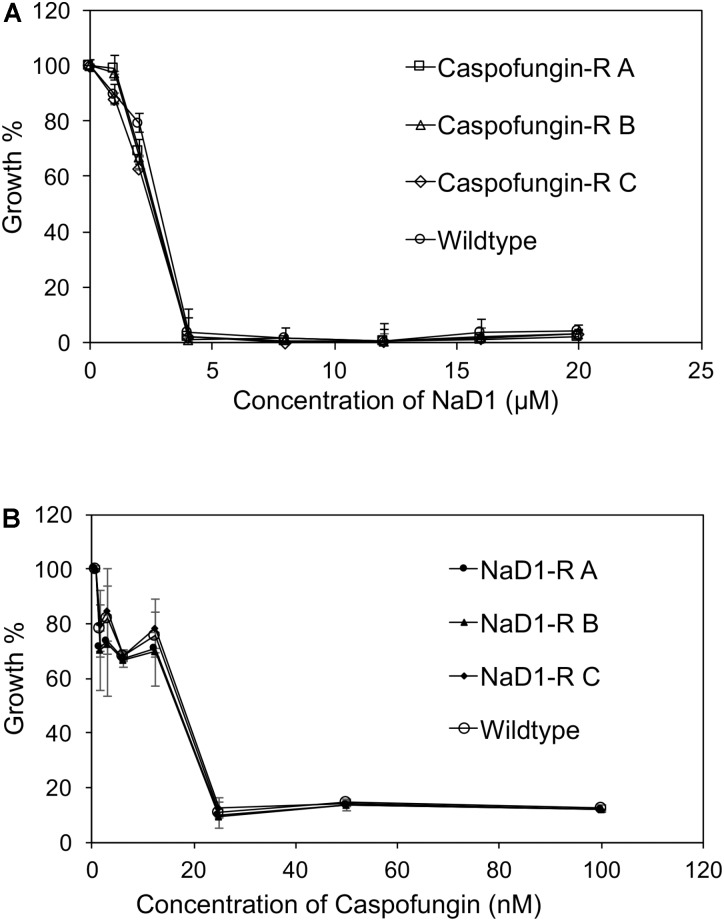
There is no cross activity between caspofungin-resistance and NaD1-resistance. Cross-resistance of caspofungin-resistant **(A)** and NaD1-resistant lines **(B)**, on NaD1 and caspofungin was determined, respectively. **(A)** Average growth percentage is shown, relative to the highest measured absorbance for each strain. Error bars represent ±standard error of the mean (*n* = 3).

The NaD1-resistant lines were tested against a range of antimicrobial molecules to determine if the observed resistance was broad-spectrum or specific to NaD1. The caspofungin-resistant strains were as sensitive to NaD1 as the wild type (**Figure 2A**), and similarly, the NaD1-resistant strains were as sensitive to caspofungin as the wild-type strain (**Figure 2B**).

**FIGURE 3:**
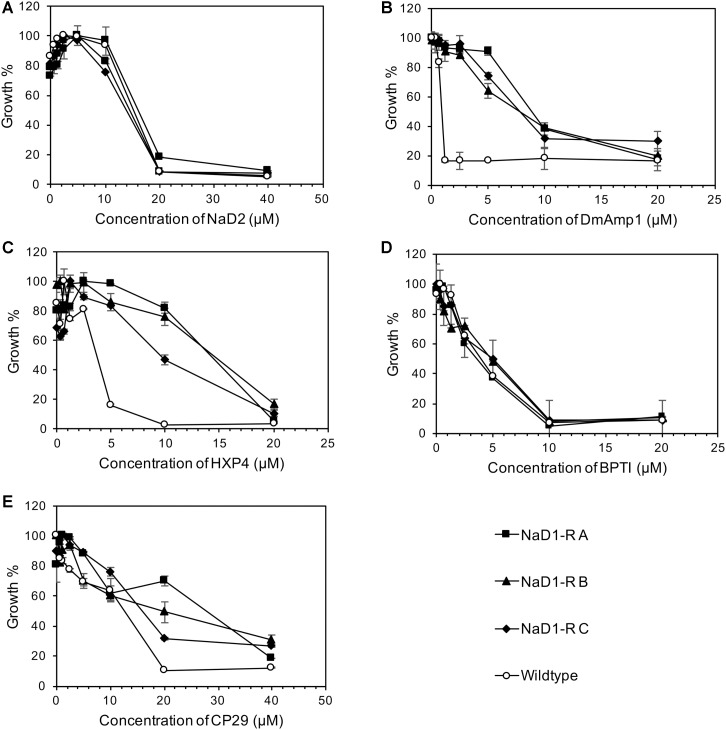
Resistance to NaD1 is not broad spectrum. Growth inhibition of NaD1-resistant strains by a selection of antimicrobial peptides of different origin and mechanisms of action. The peptides examined were: the plant defensins NaD2 **(A)**, DmAMP1 **(B)**, and HXP4 **(C)**, BPTI, a trypsin inhibitor from *Bos Taurus* **(D)**, and the insect-derived cercropin variant CP29 **(E)**. NaD1-resistant strains were also resistant to the plant defensins HXP4 and DmAMP1 but not to NaD2 or the antifungal BPTI. The three NaD1-resistant strains were slightly more resistant to CP29. Average growth percentage is shown, relative to the highest measured absorbance for each strain. Error bars represent ±standard error of the mean (*n* = 3).

NaD1-resistant strains were tested against some other plant defensins; NaD2 from *Nicotiana alata*, DmAmp1 from *Dahlia mercki* and the chimeric defensin HXP4. The NaD1-resistant strains were not resistant to NaD2 with an MIC of 20 μM, which was the same as the wild-type (**Figure 3A**). However, they were more resistant to DmAMP1 with an MIC of 20 μM compared to 1.25 μM for the wild type (**Figure 3B**) and to HXP4 with an MIC of 20 μM compared to 10 μM for the wild type (**Figure 3C**). Similarly, NaD1-resistant strains were not resistant to two unrelated cationic antifungal proteins, bovine pancreatic trypsin inhibitor (BPTI) and the insect cecropin CP29. The MIC for BPTI against both the wild type and the NaD1-resistant cultures was 10 μM (**Figure 3D**). When incubated with CP29 the NaD1-resistant strains grew slightly better than wild-type at concentrations below the MIC, but the MIC was the same for all strains tested (**Figure 3E**).

### There Is a Fitness Penalty Associated With NaD1 Resistance

**FIGURE 4:**
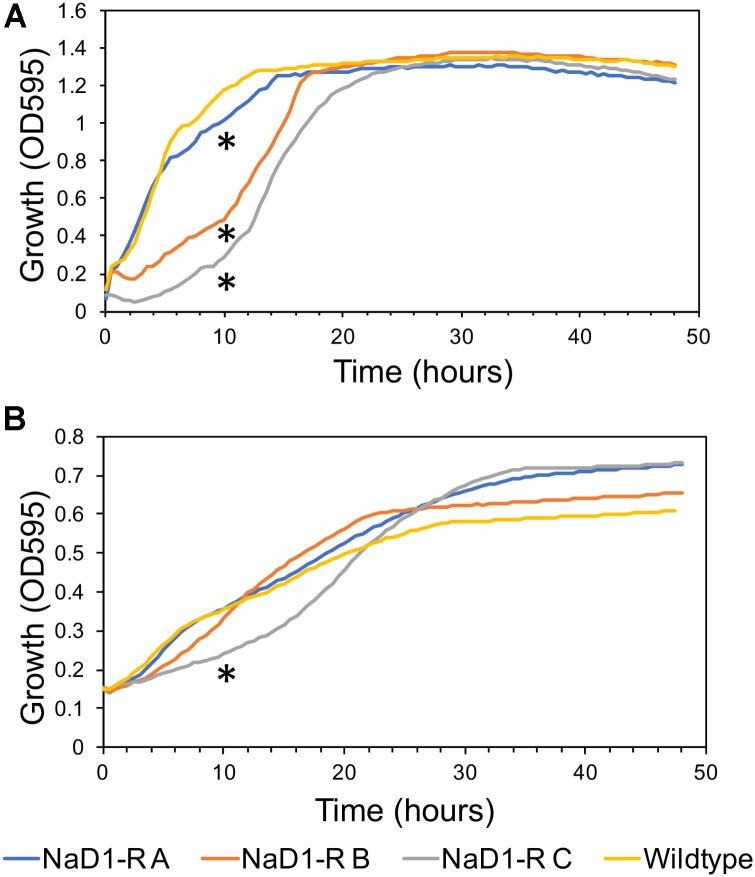
Growth rate of NaD1-resistant strains in YPD and ½ PDB medium compared to wild-type *S. cerevisiae* BY4741. Growth rate in the absence of antifungals was determined for NaD1-resistant strains in YPD medium **(A)** and ½ PDB medium **(B)**. An asterisk (^∗^) denotes a statistically significant difference in growth rate compared to wild-type, by a two-tailed homoscedastic *T*-test (*P* < 0.05, *n* = 6).

**FIGURE 5:**
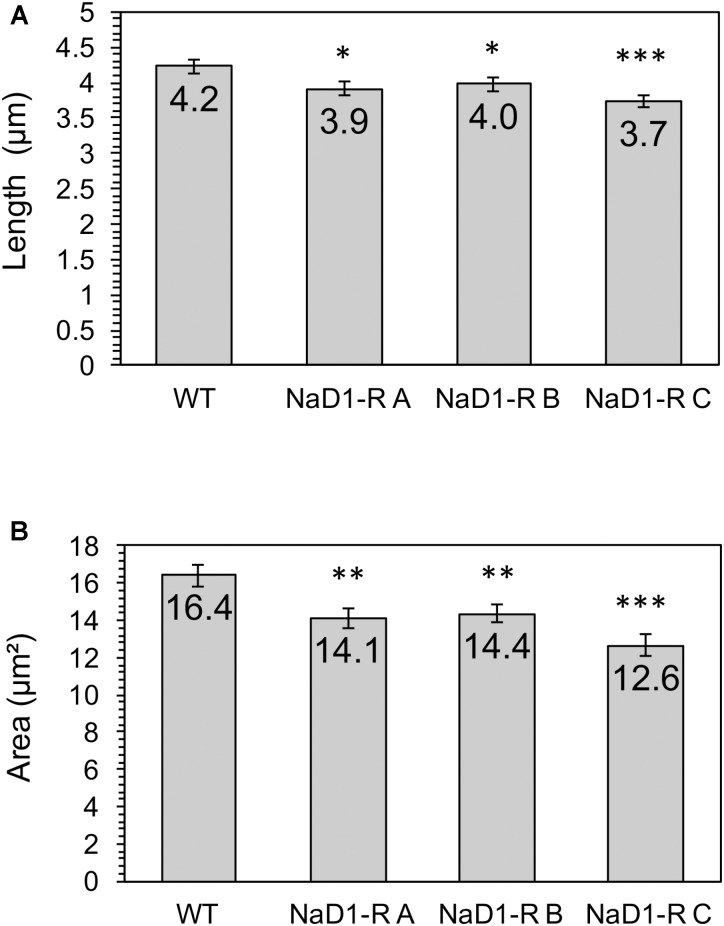
NaD1-resistant strains are smaller in length and area compared to wild type. Cross-sectional length **(A)** and area **(B)** was determined for NaD1-resistant strains and wild-type. Average values are shown ±1 standard error of the mean (*n* = 30). P value determined by two-tailed homoscedastic *T*-test indicated by ^∗^*P* < 0.05, ^∗∗^*P* < 0.01, ^∗∗∗^*P* < 0.001.

The relative fitness of the NaD1-resistant strains was assessed by comparing growth rate over 48 h in two different growth media. NaD1-resistant strains B and C grew slower in YPD for the first 18 h but reached the same culture density as wild-type after 23 h. NaD1-resistant strain A grew marginally slower than the wild type (**Figure 4A**). The growth in ½ PDB was less varied, with only NaD1-resistant strain C growing significantly more slowly than wild-type (**Figure 4B**). The cellular dimensions of NaD1- resistant strains were smaller than the wild type in both length and area (**Figure 5**).

### NaD1-Resistant Strains Are Sensitive to Cell Wall Stressors and Are Resistant to Osmotic Stress

**FIGURE 6:**
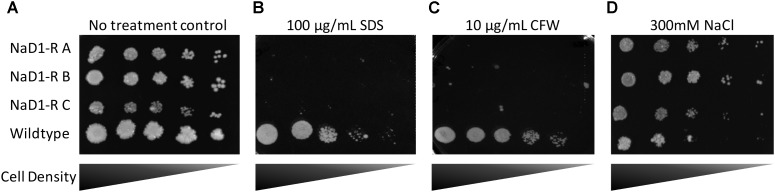
NaD1-resistant strains are sensitive to SDS and calcofluor white (CFW), but are resistant to NaCl. NaD1-resistant and wild-type *S. cerevisiae* BY4741 cells were diluted and spotted onto YPD agar alone **(A)** or YPD supplemented with SDS **(B)**, CFW **(C)**, or NaCl **(D)**. NaD1-resistant strains but not the wild type were inhibited by 100 μg/mL SDS or 10 μg/mL CFW compared to wild type. However, NaD1-resistant strains maintained growth at concentrations of NaCl that the wild type strains could not tolerate. Images are representative of three repeated experiments, all showing similar results.

Potential alterations to the cell wall and membrane were examined by exposing the NaD1 resistant strains to SDS and CFW. SDS is an anionic detergent that causes cell wall stress, and membrane permeabilization and CFW is a cell wall stressor that binds to chitin. This revealed a significant growth defect of the NaD1-resistant strains in the presence of SDS or CFW (**Figures 6B,C**). Sensitivity of the NaD1-resistant strains was observed at 12.5 μg/mL SDS (Supplementary Figure 3) and at 1 μg/mL CFW (Supplementary Figure 4).

In *Candida albicans*, the HOG1 osmotic stress response pathway is involved in tolerance to NaD1 (Hayes et al., 2013). It was, therefore, important to assess whether the *S. cerevisiae* NaD1-resistant strains had an altered osmotic stress response. NaD1-resistant strains grew better than wild-type at 200 mM NaCl (**Figure 6D** and Supplementary Figure 5). This supported the hypothesis that NaD1-resistance correlates with increased osmotic stress tolerance.

### NaD1-Resistant Strains Are Not Resistant to Hydrogen Peroxide, UV Light, or Heat

**FIGURE 7:**
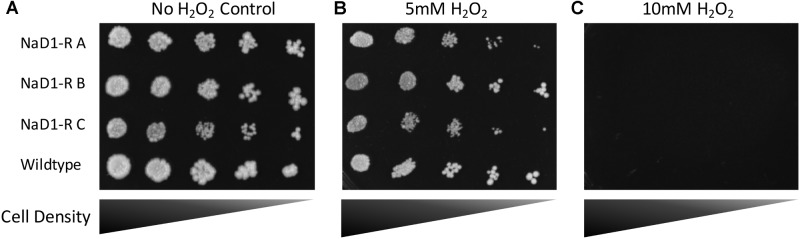
NaD1-resistance does not alter sensitivity to oxidative stress. NaD1-resistant and wild-type *S. cerevisiae* BY4741 cells were diluted and spotted onto YPD agar containing 0 mM **(A)**, 5 mM **(B)**, or 10 mM **(C)** H_2_O_2_. There was no difference in H_2_O_2_ sensitivity between the NaD1-resistant strains compared to the wild type. Images are representative of three experiments, all showing similar results.

NaD1 induces ROS production in *Candida albicans*, which is a contributing factor to cell death. However, at low NaD1 levels, *C. albicans* cells cope by activation of the HOG1 pathway and enhancing transcription of genes that protect against oxidative stress (Hayes et al., 2013). Thus, the NaD1-resistant strains were tested for sensitivity to hydrogen peroxide generated oxidative stress. The NaD1-resistant strains grew the same as the wild-type strain in the presence of a range of hydrogen peroxide concentrations (**Figure 7** and Supplementary Figure 6).

NaD1-resistant strains were also tested for resilience to ultraviolet light (UV) that causes DNA damage, as well as their resilience to heat shock.

There was no observable difference in the growth of the NaD1-resistant strains and the wild-type cells after UV light or heat treatment (Supplementary Figure 7).

### Genetic Characterization of NaD1 Resistance

The genomes of each of the NaD1-resistant and non-selected control lines were sequenced to identify mutations exclusively found in NaD1 resistant lines. Mutated genes identified in the resistant isolates were compared to the genes in the non-selected wild type (**Table 2**), along with the predicted amino acid changes. There were eight mutated genes found in NaD1-resistant strain A, five mutated genes in strain B, and seven genes mutated in strain C. There were three genes mutated in all three strains (FPS1, TOM1, and RSP5) and two genes were mutated in both NaD1-resistant B and C strains (PHO84 and CWP2) (**Table 2**). The results obtained from the VEP (McKenna et al., 2010), which determines the consequence of DNA variants on protein sequence, are listed in **Table 2**. A description of predicted functions for the affected genes is listed in **Table 3**.

**Table 2:** Summary of variants that disrupted protein coding regions in NaD1-R strains.

Gene Name	Amino acid change	NaD1-R strains containing variant	Type	Inference
BUD4	p.Asn415Asp	A	SNV	Tolerated missense variant
CWP2	p.Leu92del	B, C	INDEL	Disruptive in-frame deletion
FPS1	p.Phe555fs	A, B, C	INDEL	Disruptive frame shift
MRPS16	p.Pro45Gln	C	SNV	Deleterious missense variant
PHO84	p.Ser183Phep.Val202Ile	BC	SNV	Deleterious missense variant
PMR1	p.Val170Ile	A	SNV	Deleterious missense variant
RAS2	p.Asp112Gly	C	SNV	Deleterious missense variant
RET2	p.Gln12His	A	SNV	Missense variant
RSP5	p.Gly689Cys	A, B, C	SNV	Missense variant
SIR3	p.Glu451^∗^STOP	A	SNV	Disruptive premature stop
SKY1	p.Trp173Leu	A	SNV	Deleterious missense variant
TOM1	p.Ala2381Gly	A, B, C	SNV	Deleterious missense variant


*The observed changes to protein coding regions of NaD1-R strains are shown along with the inferred impact on the encoded protein. SNV, single nucleotide variant; INDEL, insertion or deletion. These genes may be viewed on the Saccharomyces Genome Database www.yeastgenome.org(Cherry et al., 2012; Engel et al., 2014).*

**Table 3:** Summary of gene functions impacted by NaD1-resistance.

Gene	Functional group	Description
BUD4	Cell wall	Protein involved in bud-site selection.
CWP2	Cell wall	Cell wall mannoprotein.
FPS1	Transport	Aquaglyceroporin, plasma membrane channel.
PHO84	Transport	Inorganic phosphate transporter.
PMR1	Transport	Calcium and manganese transport to the Golgi.
SKY1	Signaling	Regulating cation homeostasis.
RAS2	Signaling	Regulates sporulation and filamentous growth.
TOM1	Ubiquitin ligase	E3 ubiquitin ligase (Hect-domain class)
RSP5	Ubiquitin ligase	E3 ubiquitin ligase (NEDD4 family)
SIR3	Chromatin binding	Chromatin remodeling.
RET2	Unknown	Retrograde transport between Golgi and ER.
MRPS16	Ribosome structure	Mitochondrial ribosomal protein.


*The genes that have mutations linked to NaD1 resistance and the description of their role in S. cerevisiae are shown. Gene ontology functional analysis revealed that some of these genes can be grouped by location or function, including: cell wall, transporter, signaling or ubiquitin ligase categories. These genes may be viewed on the Saccharomyces Genome Database www.yeastgenome.org (Cherry et al., 2012; Engel et al., 2014).*

### Determining the Relative Contributions of Loss of Function Mutations to NaD1 Tolerance

It was considered likely that most of the observed mutations would have resulted in a loss-of-function phenotype for the affected genes. To test this hypothesis, strains with single-gene knockouts for mutated genes were retrieved from the yeast deletion set (Winzeler et al., 1999) and antifungal growth assays were performed to assess whether gene deletion replicated the NaD1-resistant phenotype. The knock-out strains were only selected from non-essential genes. The antifungal assay revealed that none of the single gene knockout mutants (*fps1*Δ, *cwp2*Δ, *mrps16*Δ, *pmr1*Δ, *pho84*Δ, and *sky1*Δ) were as resistant to NaD1 as the three NaD1 resistant strains. Instead, each of the knockout mutations conferred partial resistance to NaD1. The highest level of resistance from a single knock-out occurred with *fps1*Δ, which had an MIC of 9 μM. Compared to the original NaD1-R mutants that had MICs of 40 μM, *cwp2*Δ, *pmr1*Δ, *mrps16*Δ, and *pho84*Δ contributed a smaller amount of resistance with an MIC of 6–7.5 μM while *sky1*Δ had the same MIC as the wild-type and control strains (**Table 4**).

**Table 4:** Comparison of NaD1 activity against single-gene deletion strains representing key resistance variants.

Strain of *S. cerevisiae*	NaD1 MIC (μM)	95% CI ±
Wild type	4.5	0.03
NaD1-resistant strain A	40	0.03
NaD1-resistant strain B	40	0.04
NaD1-resistant strain C	40	0.03
Control A	4.5	0.03
Control B	4.5	0.03
Control C	4.5	0.03
FPS1 knockout	9	0.05
CWP2 knockout	6	0.03
MRPS16 knockout	7.5	0.06
PMR1 knockout	6	0.03
PHO84 knockout	6	0.04
SKY1 knockout	4.5	0.02


*The minimum inhibitory concentration (MIC) of NaD1 was determined for single gene deletion strains linked to NaD1 resistance is shown, alongside control lines and the NaD1-resistant strains. Average values and a 95% confidence interval (CI) were calculated from three independent experiments.*

## Discussion

### Resistance to NaD1 Is Slow to Develop

Antimicrobial peptides represent a promising next generation of therapeutics to combat drug-resistant fungi and bacteria (Wang et al., 2016). Peptides provide benefits as pharmaceuticals over small molecule drugs because they bind with high specificity to their targets and require a relatively large interaction interface, which results in fewer off-target side effects (Craik et al., 2013). Plant defensins are known to bind to lipids and polysaccharides (Kvansakul et al., 2016; Payne et al., 2016; Poon et al., 2014). As hypothesized in this report, resistance to the antifungal peptide NaD1 developed more slowly than resistance to the small molecule drug caspofungin (**Figure 1**). The MIC of NaD1-resistant strains was only 10-fold greater than wild type, which was less than the equivalent caspofungin-resistant strains (20-fold greater than wild type) and caspofungin resistance developed more rapidly than NaD1 resistance (**Figure 1**). Our observation is consistent with the reported benefits of peptide drugs, where their larger interaction surface requires more changes to the target before binding is disrupted.

### Resistance to NaD1 Did Not Confer Broad-Spectrum Resistance to Other Antifungal Peptides

An example of broad-spectrum resistance to cationic AFPs has been reported for an *agp2*Δ mutant of *S. cerevisiae* whereby resistance was mediated by an accumulation of positive charges at the cell surface that repelled positively charged antifungal peptides (Bleackley et al., 2014b). Therefore, it was important to determine whether evolved NaD1-resistant strains were resistant to other cationic peptides. The NaD1-resistant strains were resistant to the plant defensins HXP4 and DmAMP1 (**Figures 3B,C**). HXP4 is a chimera of NaD1 and NaD2, with a similar mechanism of action of NaD1, and hence was expected to share cross-resistance with NaD1 (Bleackley et al., 2016). DmAmp1 a plant defensin from *Dahlia merckii*, has a different mechanism of action to NaD1 (Parisi et al., 2018) whereby it binds to sphingolipids in the cell wall and plasma membrane of *S. cerevisiae* to exert antifungal activity (Thevissen et al., 2000). Although DmAMP1 and NaD1 have different mechanisms of action, they each stimulate the high-osmolarity glycerol (HOG) pathway in *C. albicans* (Hayes et al., 2013) and mutants in that pathway (*hog1* or *pbs2*) were more sensitive to NaD1 and DmAmp1. In *S. cerevisiae*, the alteration of the osmotic stress pathway could also affect the sensitivity to DmAmp1. The antifungals BPTI and NaD2 were still effective against the NaD1-resistant strains demonstrating the developed resistance was not broad spectrum (**Figures 3A,D**). BPTI inhibits *S. cerevisiae* growth by targeting a magnesium transporter and blocking the uptake of magnesium. Therefore, it was expected that the NaD1-resistant strains would still be sensitive to BPTI (Bleackley et al., 2014a). The mechanism of action of NaD2 is mostly unknown, but it is known to bind to phosphatidic acid to exert its antifungal activity, unlike NaD1 that binds to both PIP2 and PA (Bleackley et al., 2016; Payne et al., 2016). The cationic peptide CP29 was less effective at sub MIC concentrations, but there was no shift in MIC detected (**Figure 3E**). Taken together this means that the resistance to NaD1 did not occur through a broad-spectrum resistance mechanism against all cationic AFPs. Plant defensins act synergistically with the clinical antifungal caspofungin and boost overall antifungal activity (van der Weerden et al., 2014; Vriens et al., 2015, 2016). We found that NaD1 was still effective against strains resistant to caspofungin (**Figure 2A**). Plant defensins may provide a very robust therapy if delivered in combination with existing clinical antifungals.

### Resistance to NaD1 Has a Fitness Penalty

NaD1-resistant strains were tested for physical differences with wild type cells, to establish whether there is a fitness penalty associated with NaD1-resistance. The cell growth assays (**Figure 4**) revealed that NaD1-resistant strains grew more slowly than the wild type strain in the rich medium, YPD. NaD1-resistant strain C grew the slowest in YPD medium, this may be due to the mutation in MRPS16, which is a mitochondrial ribosomal protein and RAS2, which regulates sporulation and filamentous growth. Knockout mutants of MRPS16 have been reported to have decreased vegetative and respiratory growth (Orij et al., 2012; Schlecht et al., 2014). A knockout of RAS2 has also been reported to have decreased fitness in YPD medium (Qian et al., 2012), supporting our observation that the NaD1-resistant strain C had the largest fitness defect. Interestingly, the growth of the NaD1-resistant strains was equivalent to wild type in ½ PDB, supporting the veracity of the antifungal assays that were all performed in this medium. Individual cells of the NaD1-resistant strains were smaller in length and cross-sectional area compared to wild-type cells grown in YPD (**Figure 5**). NaD1-resistant strains were consequently tested against a range of cell wall stressors to investigate whether adaptations to NaD1 resistance had altered the properties of the cell wall and membrane. SDS is a detergent with a negatively charged head group that is commonly used to test the susceptibility of yeast cells to membrane permeabilization and cell wall perturbation (Sirisattha et al., 2004; Gao et al., 2014). NaD1-resistant strains were more sensitive to SDS than the wild type strain (**Figure 6**). This sensitivity suggests that strains with enhanced tolerance to NaD1 have modifications their cell walls or plasma membranes, and may be more susceptible to alternative antifungal drugs. NaD1-resistant strains were also sensitive to CFW, which binds to cell wall chitin and leads to permeabilization (**Figure 6**). NaD1-resistant strains may contain more chitin in their cell wall and therefore increase the binding of CFW (Roncero et al., 1988). Furthermore, CFW relies on a functional Hog1 pathway for its antifungal activity, and thus hyperactive Hog1 signaling to protect against defensin activity may result in heightened sensitivity to CFW (García-Rodriguez et al., 2000). NaD1-resistant strains were also tested against more diverse environmental stresses, but we found no evidence for protection or sensitivity to oxidative stress (**Figure 7**), DNA damage or heat stress. We have established a link between NaD1 resistance and cell wall stress. The observed NaD1 resistance appears limited to the NaD1 mechanism of action, and it does not mitigate oxidative stress, DNA damage or heat shock.

### Unlike Resistance to Azoles and Echinocandins, Resistance to NaD1 Occurs via Multiple Quantitative Mutations

Whole genome sequencing revealed multiple genes were linked to NaD1 resistance (**Table 2**). The functional diversity of these genes revealed that the mechanism of NaD1 is likely to involve more than a single protein target. This contrasts with resistance to caspofungin, which can be achieved by a single amino acid alteration in the targeted β-glucan synthase Fks1p (Katiyar and Edlind, 2009), or resistance to fluconazole with mutations to the Erg11p enzyme (Sionov et al., 2012). Our caspofungin-resistant mutants all followed this path to resistance, with each acquiring a single mutation at residue 639 of Fks1p. The NaD1-resistant strains acquired mutations related to the protection from osmotic stress, alteration of the cell wall, solute transport, signaling, and cation homeostasis (**Table 3**). In summary, unlike echinocandin and azole classes of fungicides, resistance to NaD1 did not feature a “hot-spot” for genomic mutations.

The NaD1-resistant strains had accumulated several mutations, and thus no single gene could be identified that was responsible for the resistance phenotype. The relative contribution of each observed mutation was assessed by comparing the level of NaD1 resistance in strains with knockouts of individual genes (**Table 4**). None of the single gene knockouts produced the level of NaD1 resistance obtained in the evolved strains. The *FPS1* knockout had the biggest effect and was mutated in all three of the evolved resistant strains. *PHO84*, *PMR1*, and *CWP2* deletion mutants contributed relatively smaller degrees of NaD1 resistance. In *PHO84* and *PMR1*, mutations in the NaD1-resistant strains were single nucleotide changes with conservative effects; it may be that protein function was only mildly affected. Combinations of mutations were not assessed as we felt that an exhaustive account of these variants was not supported as we did not have enough individual strains to support which combination was evolutionarily more successful. Future work will focus on increasing the number of individual resistant lines studied. This should provide quantitative data on the relative benefit of different combinations of variants.

There were SNV’s found in the TOM1 and RSP5 genes of all three NaD1-resistant strains. The SNV’s are unlikely to lead to a complete loss of function and instead are likely to represent a partial loss or gain of function. Both TOM1 and RSP5 are E3 ubiquitin ligases, a class of protein that tags protein substrates for destruction. Ubiquitin ligases regulate diverse functions including cell trafficking, DNA repair, and signaling. TOM1 regulates mRNA export from the nucleus and targets excess histones for degradation (Saleh et al., 1998; Singh et al., 2009). RSP5 is an essential gene that regulates a variety of processes including mitochondrion organization and sorting of multivesicular bodies (Katzmann et al., 2004; McNatt et al., 2007; Kaliszewski and Zoladek, 2008). In *C. albicans*, NaD1 is known to cross the plasma membrane via endocytosis. It is possible that a restriction of multivesicular transport could also restrict NaD1 movement inside the target cell. It has also been reported that decreased function of RSP5 can increase the susceptibility to cell wall stressors such as calcofluor, as was seen for NaD1-resistant lines in our stress assays (**Figure 6**). The identification of genomic variants in essential genes highlights an advantage of natural selection and genome sequencing as a method to identify mechanisms of resistance mechanisms.

### Resistance to NaD1 Has a Common Theme of the Osmotic Stress Response

In our study, *FPS1* was mutated in all three of the evolved NaD1-resistant strains. *FPS1* encodes an aquaglyceroporin plasma membrane channel with a role in the efflux of glycerol and xylitol (Luyten et al., 1995). This efflux pump maintains osmotic balance by moderating the passive diffusion of glycerol (Toh et al., 2001). NaD1-resistant strains all contained a frameshift (Phe555fs) that prevents translation of 115 amino acids from the C-terminal regulatory domain of the FPS1 protein (Hedfalk et al., 2004). This could result in substantial modification to its function and cellular osmotic balance because this 115-amino acid region contains seven phosphorylation sites and two ubiquitinylated lysine sites that regulate the function of the channel. It is unclear if loss of this c-terminal region would cause protein instability and a total loss of function, or if it would produce an unregulated glycerol channel. The phenotype of the FPS1 knockout had significant resistance to NaD1, suggesting loss of function is the most likely result of the frameshift mutation. Fps1p is regulated by the HOG pathway in *S. cerevisiae*. In wild-type cells the Fps1p-mediated efflux of glycerol decreases when the cell is under hyper-osmotic (high salt) stress which in turn increases the internal accumulation of glycerol (Hedfalk et al., 2004). In theory, the *FPS1* deletion mutants will be resistant to hyper-osmotic shock as they are always accumulating intracellular glycerol (Toh et al., 2001). This resistance to hyper-osmotic stress was confirmed in the NaCl spot assays where we observed increased growth of NaD1-resistant strains under high salt conditions compared to wild-type cells (**Figure 6D**). We hypothesize that loss of FPS1 activity would prevent the release of excess turgor pressure via glycerol efflux, and result in excess pressure on the cell wall and susceptibility to cell wall stress. This is supported by previous reports that show that a combination of a FPS1 deletion with cell wall weakening mutations in *S. cerevisiae* results in cell lysis and lethality (Tamás et al., 1999). In work by García-Rodriguez et al. (2000), the ability of CFW to inhibit *S. cerevisiae* was dependent on a functional HOG pathway (**Figure 7C**). The work of Hayes et al. (2013) in *Candida albicans* supports this model as NaD1 is known to activate the osmotic stress response, or HOG, pathway in *C. albicans* and permit tolerance of low amounts of NaD1. In addition, *hog1* mutants are more sensitive to NaD1 and DmAmp1 (Hayes et al., 2013). In a similar mechanism, via modification of the osmotic balance of the cell, our yeast mutants gained resistance to plant defensins NaD1 and DmAMP1 and conversely increased their sensitivity to cell wall stressors. The role of *FPS1* in resistance to NaD1 is consistent with NaD1 activation of Hog1p in *C. albicans*, as FPS1p activity is regulated by Hog1p in *S. cerevisiae* (Lee et al., 2013; Muir et al., 2015). One possible mechanism for *FPS1*-mediated NaD1 resistance is that *FPS1* mutants accumulate high intracellular concentrations of glycerol, which stabilizes lipid bilayers and protects the cellular organelles that are targeted by the NaD1 protein.

The NaD1-resistant strains also had mutations in other solute transporters. *PHO84* an inorganic phosphate transporter and low affinity manganese transporter and, *PMR1* a high affinity calcium and manganese transporter (Lapinskas et al., 1995; Jensen et al., 2003). Calcium is known to be involved in the response to osmotic stress, *S. cerevisiae* releases a stretch-activated pulse of calcium ions in response to cellular swelling from hypo-osmotic stress (Batiza et al., 1996; Tong et al., 2004). It is possible the Pmr1p transporter produces this calcium release.

**FIGURE 8:**
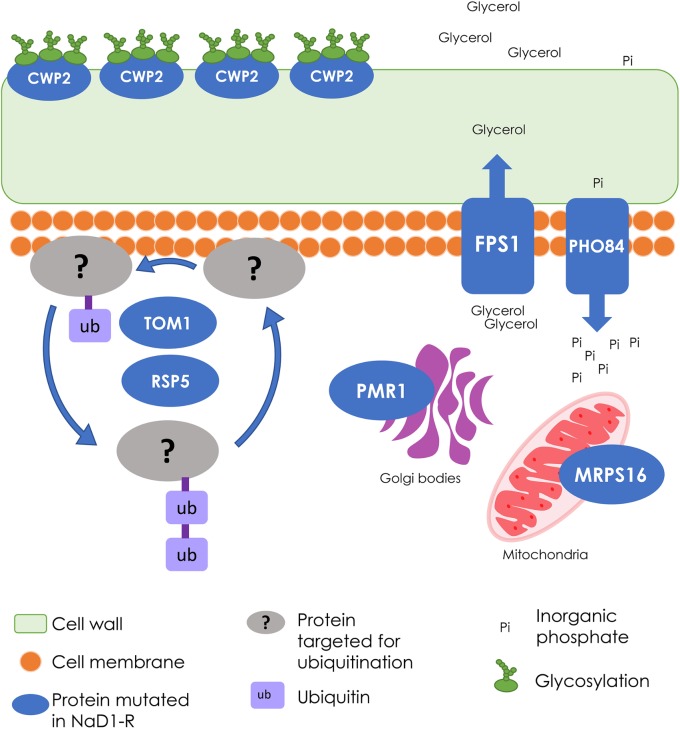
Summary of the wild-type cellular processes that were modified in NaD1-resistant strains. The cellular processes involved in NaD1 resistance included the cell wall, transport, and signaling functions. FPS1 is a glycerol transporter that protects the cell from osmotic shock by exporting or preventing export of glycerol; the FPS1 gene deletion was the most resistant single-gene knockout tested. Other gene deletions that contributed to NaD1 resistance were: CWP2, which is a cell wall mannoprotein, PHO84, which is a phosphate and manganese ion transporter, PMR1, which transports calcium and manganese to the Golgi bodies, and MRPS16, which is a mitochondrial ribosomal protein. TOM1 and RSP5 are essential ubiquitin ligases that were mutated in all NaD1-resistant strains. Ubiquitin ligases target proteins for degradation and regulate a range of processes, including multivesicular body sorting. Overall, these genes regulate a range of cellular processes with a common theme of cell wall and osmo-regulation.

NaD1-resistant strains also had mutations in genes that affect cell wall composition including *CWP2*, *RAS2*, and *BUD4*. *CWP2* encodes a mannoprotein that has a major role in stabilizing the cell wall (Frieman and Cormack, 2003). Mutants with a *cwp2* deletion are more sensitive to CFW and congo red, which are cell wall stressors, providing another explanation for why the NaD1-resistant strains were more sensitive to CFW than the wild type in **Figure 7C** (van der Vaart et al., 1995). Both *RAS2* and *BUD4* affect the structure of the cell wall and are associated with protein localization to the bud neck (Gimeno et al., 1992; Kang et al., 2013). Hence, changes in cell size and growth in the resistant strains (**Figures 5, 6**) could be linked to the mutations in these genes. In summary, the NaD1-resistant mutants were characterized by mutations that increased resistance to hyperosmotic stress and conversely increased sensitivity of the resistant strains to cell wall stressors such as CFW and SDS. An overall summary of the key changes observed in NaD1-resistant strains is presented in **Figure 8**.

## Conclusion

In this paper, we described the development of *S. cerevisiae* tolerance to an antifungal plant protein, the defensin NaD1. The overall aim was to compare the rate and mechanism of resistance development of a small protein to a small molecule antifungal of the echinocandin class. This study identified that resistance to the defensin NaD1 was slow to develop and had limited effectiveness compared to caspofungin resistance. A fitness penalty was associated with NaD1 resistance, thus if the selective pressure of NaD1 was removed it is likely that non-resistant strains would outcompete the NaD1 resistant strains. Increased tolerance to NaD1 developed via the accumulation of multiple mutations over time, and not via a single target site modification as with caspofungin. There was no cross resistance observed between NaD1 or caspofungin resistance, therefore, this study indicates that NaD1, and by extension other plant defensins, may complement existing clinical antifungals due to their resilience and unique mechanism of action.

## Author Contributions

AM performed the experiments and wrote the manuscript. MB, MA, and RL edited the manuscript and designed the experiments.

## Conflict of Interest Statement

MA is the Executive Director and Chief Scientific Officer of Hexima Ltd. The remaining authors declare that the research was conducted in the absence of any commercial or financial relationships that could be construed as a potential conflict of interest.
