# Supplementary material for: Resistance to the Plant Defensin NaD1 Features Modifications to the Cell Wall and Osmo-Regulation Pathways of Yeast
Source: Front Microbiol. 2018 Jul 24;9:1648. doi: 10.3389/fmicb.2018.01648 (PMC6066574; doi:10.3389/fmicb.2018.01648)
Supplement: Supplementary file 1 [file Data_Sheet_1.docx]

Supplementary Material

Resistance to the Plant Defensin NaD1 Features Modifications to the Cell Wall and Osmo-Regulation in Yeast

**Amanda I. McColl, Mark R. Bleackley, Marilyn A. Anderson, Rohan G. T. Lowe* Correspondence:** Corresponding Author: [r.lowe@latrobe.edu.au](mailto:r.lowe@latrobe.edu.au)


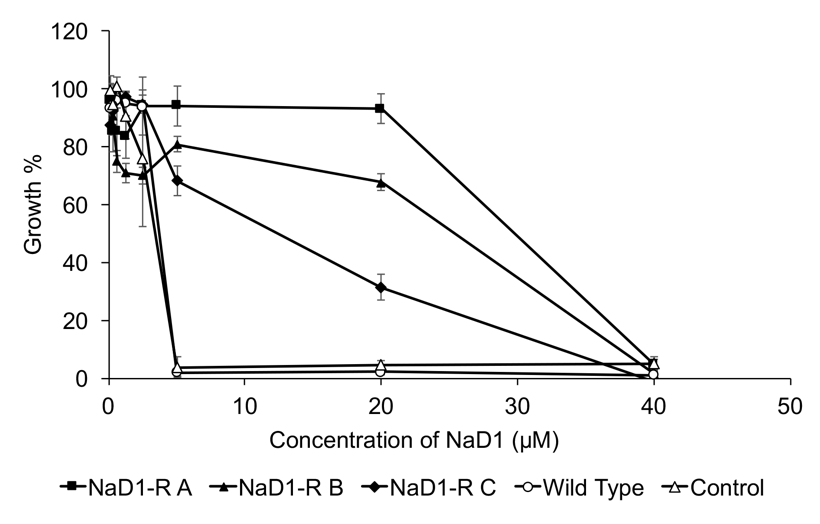


**Supplementary Figure 1.** Antifungal activity of NaD1 against NaD1-resistant isolates. Growth of NaD1-resistant isolates of *S. cerevisiae* in the presence of NaD1, compared to the wild type BY4741 strain and a control strain that was passaged 15 times in the absence of NaD1. Growth % is relative to the highest measured absorbance for each strain. Error bars represent +/- standard error of three replicates for each NaD1-resistant strain (n = 3).
