# Supplementary material for: Resistance to the Plant Defensin NaD1 Features Modifications to the Cell Wall and Osmo-Regulation Pathways of Yeast
Source: Front Microbiol. 2018 Jul 24;9:1648. doi: 10.3389/fmicb.2018.01648 (PMC6066574; doi:10.3389/fmicb.2018.01648)
Supplement: Supplementary file 2 [file Data_Sheet_2.docx]

Supplementary Material

Resistance to the Plant Defensin NaD1 Features Modifications to the Cell Wall and Osmo-Regulation in Yeast

**Amanda I. McColl, Mark R. Bleackley, Marilyn A. Anderson, Rohan G. T. Lowe* Correspondence:** Corresponding Author: [r.lowe@latrobe.edu.au](mailto:r.lowe@latrobe.edu.au)


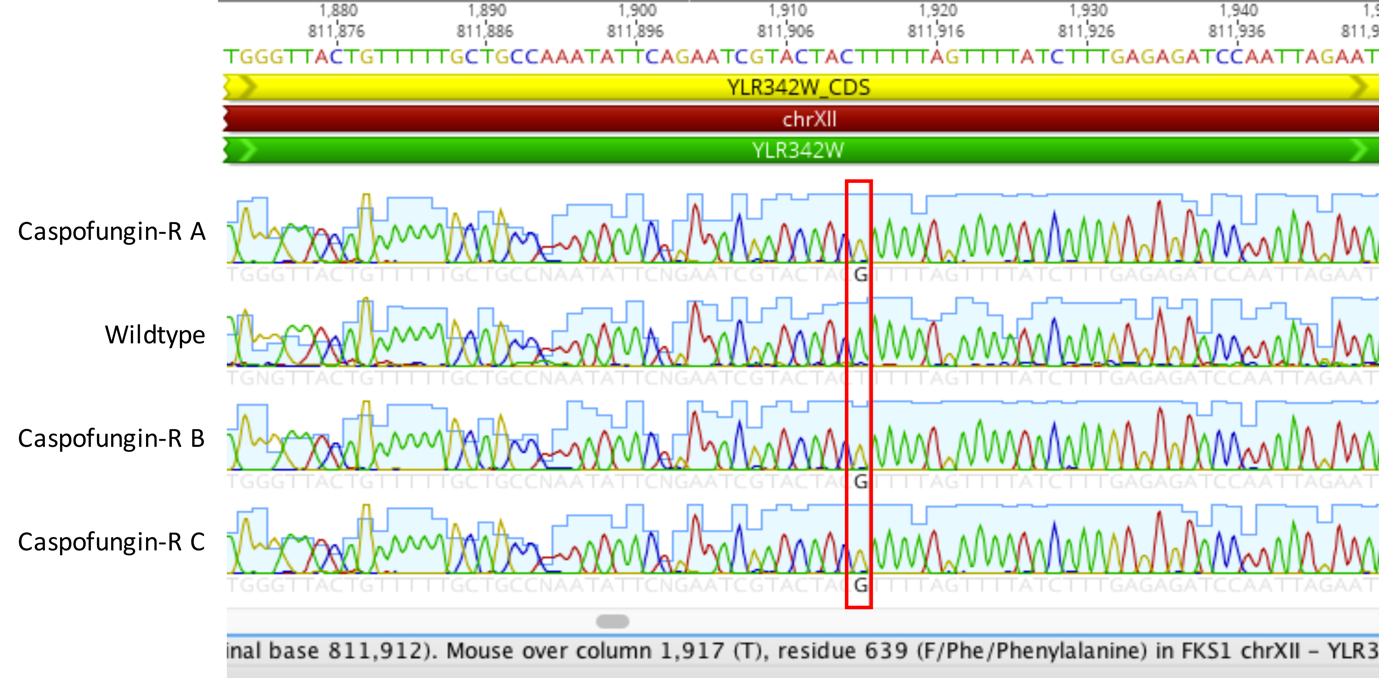


**Supplementary Figure 2.** **Sequencing of the FKS1 gene in caspofungin-resistant isolates.** Sanger sequencing data for Fks1 of caspofungin-resistant isolates. It shows the region containing a single nucleotide polymorphism (T/G) that resulted in a F639V point mutation in resistant strains that was absent in the wildtype BY4741.
