# Supplementary material for: Resistance to the Plant Defensin NaD1 Features Modifications to the Cell Wall and Osmo-Regulation Pathways of Yeast
Source: Front Microbiol. 2018 Jul 24;9:1648. doi: 10.3389/fmicb.2018.01648 (PMC6066574; doi:10.3389/fmicb.2018.01648)
Supplement: Supplementary file 3 [file Data_Sheet_3.docx]

Supplementary Material

Resistance to the Plant Defensin NaD1 Features Modifications to the Cell Wall and Osmo-Regulation in Yeast

**Amanda I. McColl, Mark R. Bleackley, Marilyn A. Anderson, Rohan G. T. Lowe* Correspondence:** Corresponding Author: [r.lowe@latrobe.edu.au](mailto:r.lowe@latrobe.edu.au)


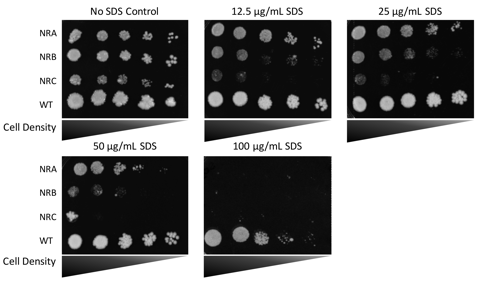


**Supplementary Figure 3.** **Titration of the SDS response of NaD1-resistant strains.** NaD1 resistant strains and wildtype *S. cerevisiae* were diluted and spotted onto YPD agar with different concentrations of SDS. NaD1-resistant strains were more sensitive SDS compared to the no treatment control and wildtype BY4741. Images are representative of 3 individual experiments. The control, and 100 μg/mL treatment are featured in figure 7 of the main text.
