# Supplementary material for: Resistance to the Plant Defensin NaD1 Features Modifications to the Cell Wall and Osmo-Regulation Pathways of Yeast
Source: Front Microbiol. 2018 Jul 24;9:1648. doi: 10.3389/fmicb.2018.01648 (PMC6066574; doi:10.3389/fmicb.2018.01648)
Supplement: Supplementary file 4 [file Data_Sheet_4.docx]

Supplementary Material

Resistance to the Plant Defensin NaD1 Features Modifications to the Cell Wall and Osmo-Regulation in Yeast

**Amanda I. McColl, Mark R. Bleackley, Marilyn A. Anderson, Rohan G. T. Lowe* Correspondence:** Corresponding Author: [r.lowe@latrobe.edu.au](mailto:r.lowe@latrobe.edu.au)


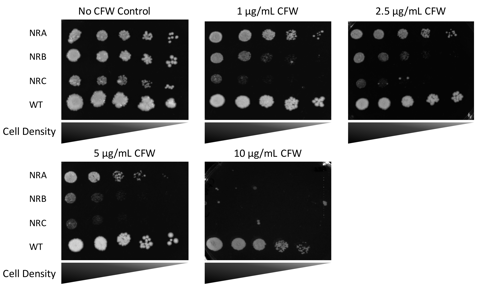


**Supplementary Figure 4.** **Titration of the calcofluor white response of NaD1-resistant strains.** NaD1-resistant strains and wildtype *S. cerevisiae* were diluted and spotted onto YPD agar with different concentrations of the chitin-binding molecule calcofluor white (CFW). NaD1-resistant strains were more sensitive to CFW compared to the wildtype. Images are representative of three individual experiments. The control, and 10 μg/mL panels are also featured in figure 6 of the main text.
