# Supplementary material for: Resistance to the Plant Defensin NaD1 Features Modifications to the Cell Wall and Osmo-Regulation Pathways of Yeast
Source: Front Microbiol. 2018 Jul 24;9:1648. doi: 10.3389/fmicb.2018.01648 (PMC6066574; doi:10.3389/fmicb.2018.01648)
Supplement: Supplementary file 5 [file Data_Sheet_5.docx]

Supplementary Material

Resistance to the Plant Defensin NaD1 Features Modifications to the Cell Wall and Osmo-Regulation in Yeast

**Amanda I. McColl, Mark R. Bleackley, Marilyn A. Anderson, Rohan G. T. Lowe* Correspondence:** Corresponding Author: r.lowe@latrobe.edu.au


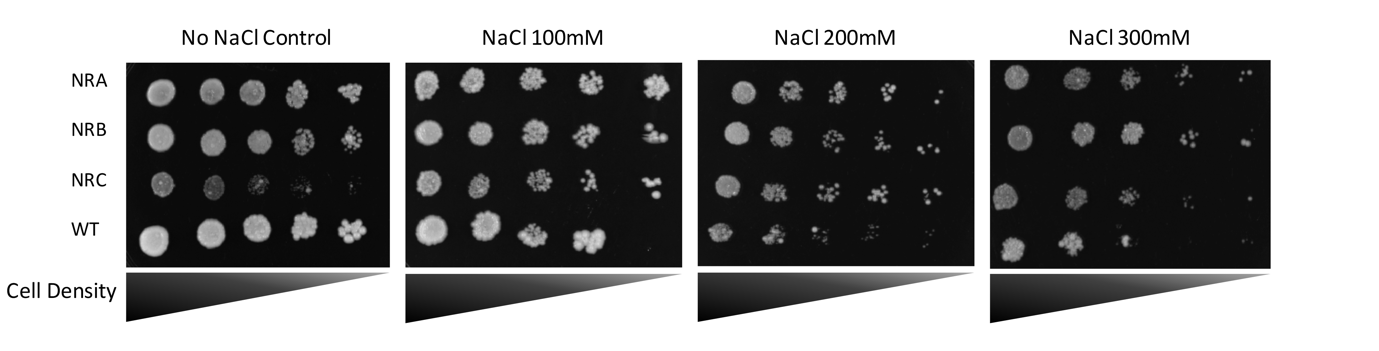


**Supplementary Figure 5.** **Titration of the NaCl response of NaD1-resistant strains**. NaD1 resistant strains and wildtype *S. cerevisiae* were diluted and spotted out on to YPD agar with different concentrations of NaCl. NaD1-resistant strains had increased resistance to NaCl compared to the wildtype. Images are representative of three individual experiments. The control, and 300 mM treatment are featured in figure 7 of the main text.
