# Supplementary material for: Resistance to the Plant Defensin NaD1 Features Modifications to the Cell Wall and Osmo-Regulation Pathways of Yeast
Source: Front Microbiol. 2018 Jul 24;9:1648. doi: 10.3389/fmicb.2018.01648 (PMC6066574; doi:10.3389/fmicb.2018.01648)
Supplement: Supplementary file 6 [file Data_Sheet_6.docx]

Supplementary Material

Resistance to the Plant Defensin NaD1 Features Modifications to the Cell Wall and Osmo-Regulation in Yeast

**Amanda I. McColl, Mark R. Bleackley, Marilyn A. Anderson, Rohan G. T. Lowe* Correspondence:** Corresponding Author: r.lowe@latrobe.edu.au


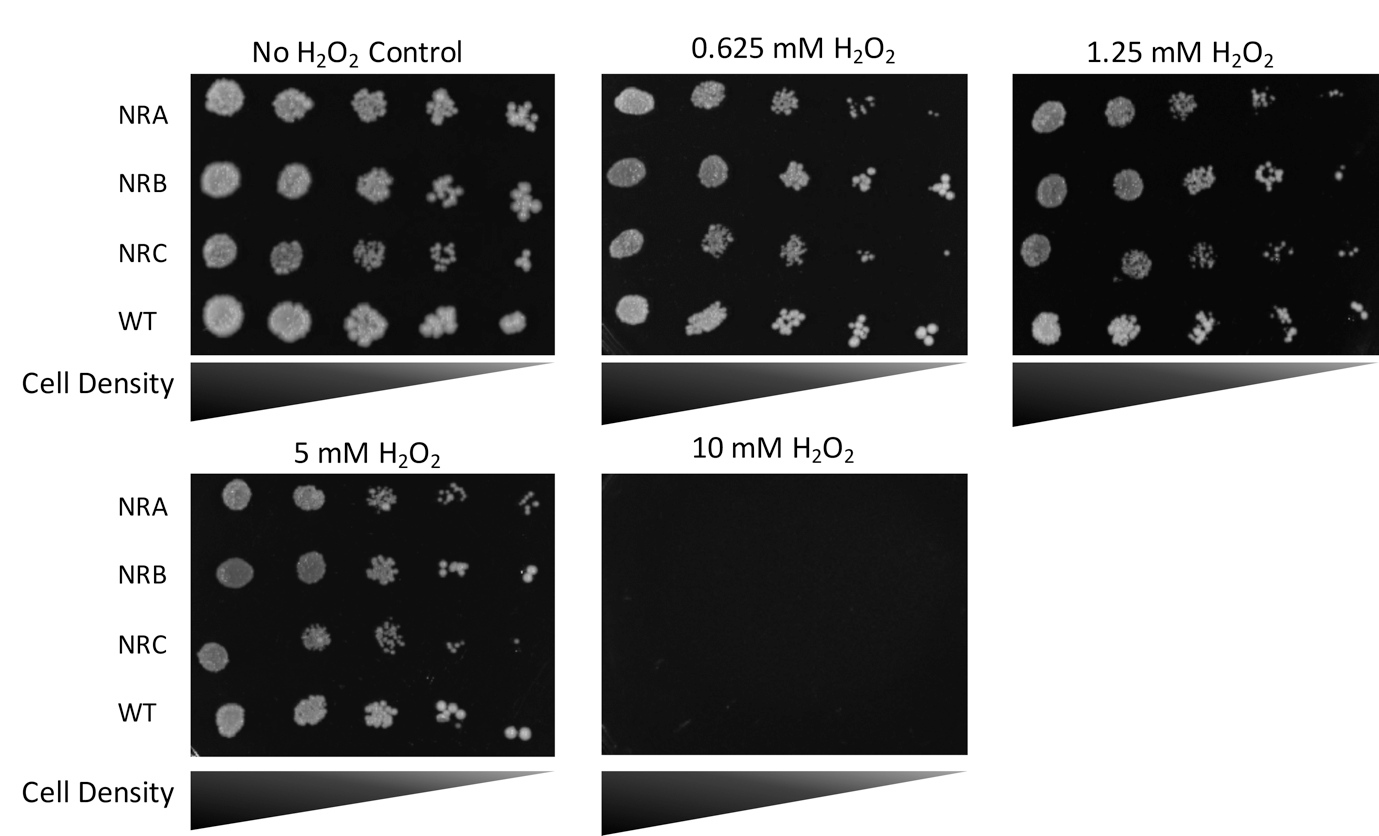


**Supplementary Figure 6.** **Titration of the hydrogen peroxide response by NaD1-resistant strains.** NaD1-resistant strains and wildtype *S. cerevisiae* cells were diluted and spotted onto YPD agar with different concentrations of hydrogen peroxide (H_2_O_2_). There was no difference in sensitivity of the NaD1-resistant strains to H_2_O_2_ compared to the wildtype. Images are representative of three individual experiments. The results for the control, 5 mM and 10 mM treatments are also shown in figure 7 of the main text.
