# Supplementary material for: Resistance to the Plant Defensin NaD1 Features Modifications to the Cell Wall and Osmo-Regulation Pathways of Yeast
Source: Front Microbiol. 2018 Jul 24;9:1648. doi: 10.3389/fmicb.2018.01648 (PMC6066574; doi:10.3389/fmicb.2018.01648)
Supplement: Supplementary file 7 [file Data_Sheet_7.docx]

Supplementary Material

Resistance to the Plant Defensin NaD1 Features Modifications to the Cell Wall and Osmo-Regulation in Yeast

**Amanda I. McColl, Mark R. Bleackley, Marilyn A. Anderson, Rohan G. T. Lowe* Correspondence:** Corresponding Author: r.lowe@latrobe.edu.au


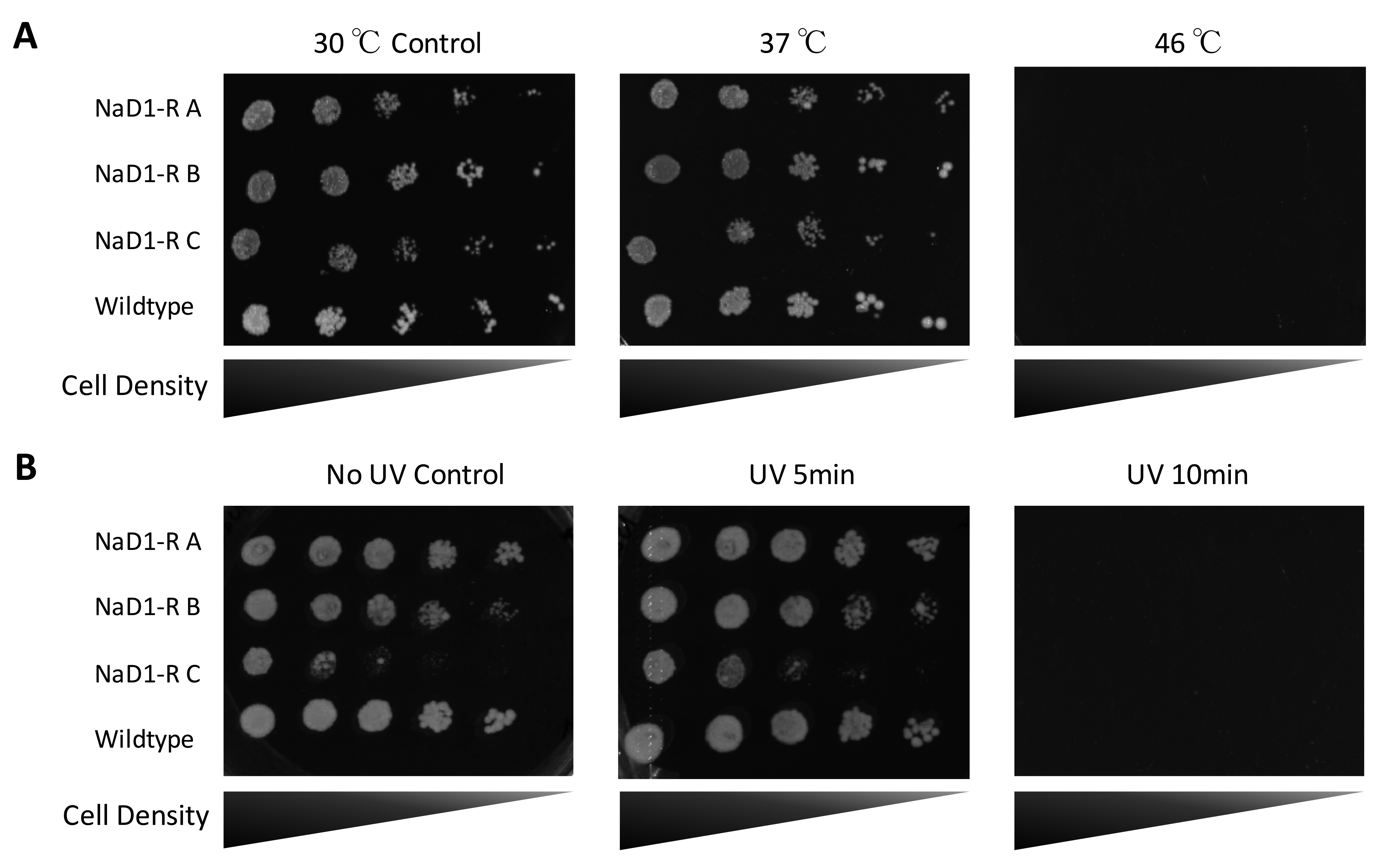


**Supplementary Figure 7.** **NaD1-resistant strains had the same sensitivity to heat stress and ultra violet light as the wild type.** (A) NaD1-resistant and wildtype *S. cerevisiae* BY4741 cells were heated at different temperatures before they were diluted and spotted onto YPD Agar. (B) NaD1-resistant and wildtype *S. cerevisiae* BY4741 cells were diluted and spotted onto YPD Agar before exposure to UV light for different times. There was no difference in the sensitivity of the NaD1-resistant strains to heat stress or UV light, compared to the controls and the wild type. Images are representative of three individual experiments.
