# Supplementary material for: Resistance to the Plant Defensin NaD1 Features Modifications to the Cell Wall and Osmo-Regulation Pathways of Yeast
Source: Front Microbiol. 2018 Jul 24;9:1648. doi: 10.3389/fmicb.2018.01648 (PMC6066574; doi:10.3389/fmicb.2018.01648)
Supplement: Supplementary file 8 [file Data_Sheet_8.docx]

Supplementary Material

Resistance to the Plant Defensin NaD1 Features Modifications to the Cell Wall and Osmo-Regulation in Yeast

**Amanda I. McColl, Mark R. Bleackley, Marilyn A. Anderson, Rohan G. T. Lowe* Correspondence:** Corresponding Author: r.lowe@latrobe.edu.au


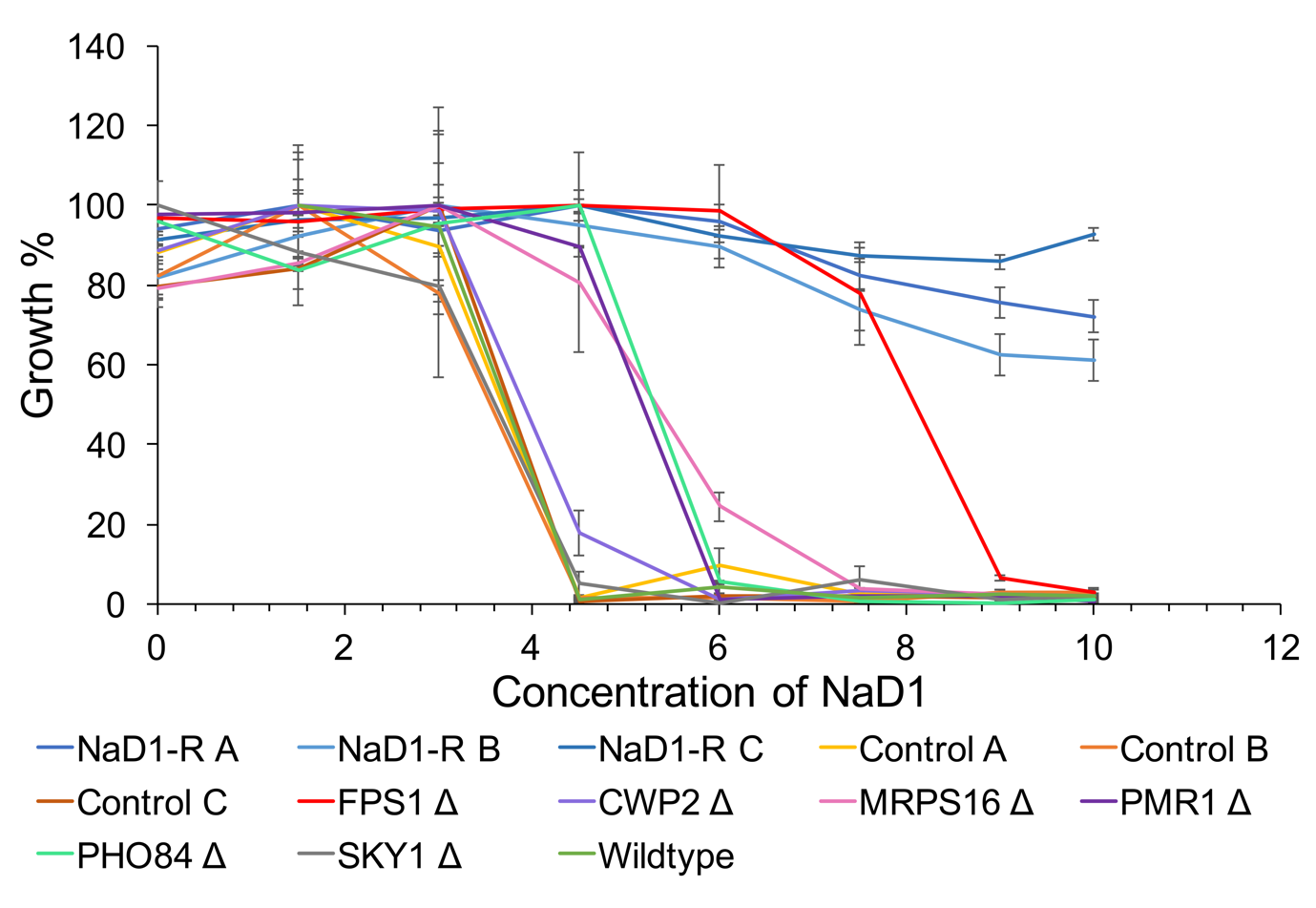


**Supplementary Figure 8.** **Determining the MIC of NaD1 for single-gene deletion strains representing key resistance variants**. Antifungal growth assays were performed for the plant defensin NaD1 against NaD1-resistant strains A, B and C, Controls A, B and C, wild-type *S. cerevisiae* BY4741 and the single-gene knock outs of the FPS1, MRP16, CWP2, PHO84, PMR1 or SKY1 genes. Average growth is plotted with standard error of the mean (n=3). Values were normalised to the maximum OD 600 nm obtained for each strain. This data is a representative example from three independent experiments.
